# The effects of intra-stomach obestatin administration on intestinal contractility in neonatal piglets fed milk formula

**DOI:** 10.1371/journal.pone.0230190

**Published:** 2020-03-23

**Authors:** Monika Słupecka-Ziemilska, Paulina Szczurek, Maria Boryczka, Małgorzata Gajewska, Piotr Wychowański, Atsukazu Kuwahara, Ikuo Kato, Żaneta Dzięgelewska, Jarosław Woliński

**Affiliations:** 1 Department of Animal Physiology, Polish Academy of Sciences, The Kielanowski Institute of Animal Physiology and Nutrition, Jabłonna, Poland; 2 Department of Animal Nutrition and Feed Sciences, National Research Institute of Animal Production, Balice, Poland; 3 Department of Physiological Sciences, Faculty of Veterinary Medicine, Warsaw University of Life Sciences, Warsaw, Poland; 4 Department of Dental Surgery, Medical University of Warsaw, Warsaw, Poland; 5 Laboratory of Physiology, Institute for Environmental Sciences & Graduate School of Nutritional and Environmental Sciences, University of Shizuoka, Shizuoka, Japan; 6 Department of Medical Biochemistry, Kobe Pharmaceutical University, Kobe, Japan; Medical University of Vienna, AUSTRIA

## Abstract

A 23-amino acid peptide named obestatin is derived from the ghrelin gene. The aim of the experiment was to study the effects of enteral obestatin administration for a 6-day period on intestinal contractility in piglets fed milk formula. Pigs were treated with 0.9% NaCl (group C) or varying doses of obestatin: 2 μg/kg body weight (BW) (group O2), 10 μg/kg BW (O10) or 15 μg/kg BW (O15) every 8 hours via a stomach tube. Blood was sampled for assessment of obestatin concentration. Duodenal and middle jejunum whole-thickness preparations were studied in an organ bath for isometric recording under electric field stimulation (EFS) and increasing doses of acetylcholine (ACh), and in the presence of atropine and tetrodotoxin (TTX). Additionally, the measurement of intestinal muscularis layer and the immunodetection of Muscarinic Acetylcholine Receptors (M1 and M2) were performed. In comparison to C animals, the obestatin concentration in blood plasma was significantly increased in groups O10 and O15. In both studied intestinal segments, significant increases in the frequency and amplitude of spontaneous contractions were observed in O15 and C groups. In the duodenum and middle jejunum significant differences in responsiveness to EFS (0.5, 5 and 50 Hz) were observed between the groups. The addition of 10^−4^ M ACh to the duodenum significantly increased the responsiveness in tissues. In contrast, in the middle jejunum a significant increase in the amplitude of contraction was observed after the addition of 10^−9^ and 10^−6^ M ACh (groups O15 and O10, respectively). Pretreatment with atropine and TTX resulted in a significant decrease in the responsiveness of the intestinal preparations from all groups, in both studied segments. The increased contractility was not dependent on the expression of muscarinic receptors. Results indicate the importance of enteral obestatin administration in the regulation of intestinal contractility in neonatal piglets.

## Introduction

Bioactive compounds in mother’s colostrum and milk are able to stimulate and regulate the development of the gastrointestinal tract (GIT) in the early postnatal period. Studies show that both the small intestine growth and maturation of its mucosa, as well as brush border enzymes activities and intestinal motility are inhibited in piglets fed milk formula compared to sow milk-fed piglets [1]. An ever-growing number of milk-derived bioactive peptides, including but not limited to, hormones, growth factors and cytokines may directly affect and support the growth and development of the newborn [2,3].

A 23-amino acid peptide named obestatin is the product of posttranslational modifications of preproghrelin, the same polypeptide that generates ghrelin [4]. The presence of significant amounts of obestatin have been previously reported in both human and rat colostrum and milk, however the physiologically relevant receptor for obestatin is yet to be discovered. Initially, G-protein-coupled receptor GPR39 was thought to be the one, nevertheless a series of studies demonstrated that obestatin is not able to bind to this receptor and to activate it [5–7]. As a physiological opponent to ghrelin, obestatin suppresses gastric emptying via inhibition of jejunal contractility and consequently decreases food intake and body-weight gain [4]. It was shown [4] that peripheral injections of obestatin reduced gastric emptying and contractile activity of rat colon muscles *in vitro*. Subsequent studies, however, did not confirm this hypothesis. In the study on fasted rats and mice it was demonstrated that peripheral administration of obestatin or co-administration with cholecystokinin failed to influence gastric motility [8]. Moreover, in adult rats there was no inhibition of gastrointestinal motor activity *in vivo* and *in vitro* by obestatin [9,10]. The literature review shows that these differences may be attributed to disparate study design and methodology (duration of the study, the route of peptide administration or the nutritional status of animals). On the contrary, Ataka et al. [11] described the inhibitory effect of intravenous obestatin injection on gastroduodenal motility of conscious rats in the fed state. Therefore, the controversy about the effect of obestatin on suppression of motility patterns in GIT continues to grow.

It should be however noted that the results described above on the obestatin effect on GIT motility were obtained in adult animals. In our recent study [12] we have however succeed to demonstrate that the effect of obestatin on bowel contractility is not only strongly age-dependent, but also specific for the segment of the intestine. In neonatal rats, an injection of obestatin significantly increased the amplitude and frequency of spontaneous contraction of whole-thickness intestinal preparations. Also, we have shown that the action and mechanisms of obestatin, even in the same animal species, were dependent on the experimental conditions (study on isolated strips vs. enteral administration).

Since substantial amounts of obestatin are present in milk, and what is more obestatin immunoreactive cells are identified in the GIT already from the 1^st^ day of life [13], this different intestinal sensitivity to obestatin in suckling animals seems to be biologically justified. These observations provide strong evidence that both endogenous and exogenous obestatin plays an important role in the modulation of GIT functioning in rat neonates. Therefore, we found it intriguing to examine the effects of enteral administration of obestatin on intestinal motor function in piglets fed milk formula.

## Materials and methods

This study was carried out in strict accordance with the recommendations in the Guide for the Care and Use of Laboratory Animals of the National Institutes of Health. The study protocol was approved by the 3^rd^ Local Ethics Committee in Warsaw, according to the Polish Law for the Care and Use of Animals (Resolution no 49/2012).

### Chemicals

Obestatin (rat-origin) was synthesized using automated Fmoc solid-phase peptide synthesis (Applied Biosystem 9030 Pioneer, Foster, CA, USA) in the Yanaihara Institute. The homology of the product was confirmed by analytical HPLC and MALDI-TOF MS. The hormone as a powder form was stored at −20°C and then dissolved in 0.9% NaCl to the final concentration just prior to use. Acetylcholine chloride (ACh), isoproterenol and atropine were acquired from Sigma-Aldrich (Germany), while tetrodotoxin (TTX) was purchased from Abcam (Great Britain).

### Animals

In total 48 male pig neonates (Polish Landrace x Pietrain) from 8 different litters were purchased from a commercial pig farm. The piglets were delivered healthy and without complications and their average birth weight was 1.50 ± 0.13 kg. For the first 24 hours after birth, the piglets were kept with their sows and then transported to the animal facility equipped with an artificial sow system (Mamina4 Special, MAZZOLARI IMPIANTISTICA, Italy). To ensure a comfortable environment for animals, the ambient temperature was decreased from 32°C to 28°C during the 6-day experimental period and a 12/12 hours light/dark cycle was provided. The piglets were housed together in four pens for approximately a 6-hour period of adaptation. Following the adaptation period, the piglets were randomly divided into four groups (n = 12). Fresh milk formula for piglets (in %: protein 19.8, fat 19.7, ash 8.2; Milky Farm, Nukamel Olen, Belgium) was distributed to each pen by means of an artificial sow system, every 75 minutes (20 times per 24 hours) in equal amounts. The daily amount of milk formula administered to piglets was calculated based on the daily body weight (BW) gain and protein intake. Piglets’ BW was recorded every morning. The amount of protein offered to neonatal pigs was progressively increased from 11.0 to 11.3 g/kg BW during the first 7 days of life. The protein concentration in the milk formula was 20%. Piglets were administered either the vehicle alone (5 ml 0.9% NaCl–group C) or obestatin (Rat obestatin, Yanaihara Institute, Japan) at a dose of 2 μg/kg BW–group O2, 10 μg/kg BW–group O10 or 15 μg/kg BW–group O15, every 8 hours via oral gavage. We used rat-origin obestatin instead of porcine, since the porcine one was neither impossible to synthetize nor commercially available. What is more, according to Green et al. [17] the amino acid homology between pig and rat obestatin is 87%. The vehicle pH was 5.8, which is optimal for obestatin bioactivity and similar to the pH of the milk formula used. Pharmacological doses of obestatin were determined on the basis of the previous *in vitro* studies on intestinal contractility [10,12]. After six days of vehicle or obestatin administrations, 6 piglets from each group were euthanized by an overdose of pentobarbiturate (Vetbutal, Biowet, Poland) and the GITs were removed for tissue sampling. From the remaining piglets, between 8^th^ and 22^nd^ day of their life, blood was collected (about 1–2 mL) for the analysis of pharmacodynamic properties of administered obestatin. After the experiment, the animals were housed for use in further research.

### Obestatin concentration

Obestatin concentration in blood serum was measured between 8^th^ and 22^nd^ day of piglet life, always 30 and 60 minutes after its administration. The concentration of obestatin in the blood samples was determined using commercial Porcine Obestatin ELISA Kit (Wuhan Fine Biological Technology Co., Ltd., Wuhan, Hubei, China, cat. no: ER0211). According to manufacturer’s instruction, the calibration curve ranged from 15.625 to 1000 pg/mL; the sensitivity was <9.375 pg/mL, and the intra-Assay and inter-Assay coefficients of variation (CVs) were <8% and <10%, respectively.

### *In vitro* studies

**Fig 1:**
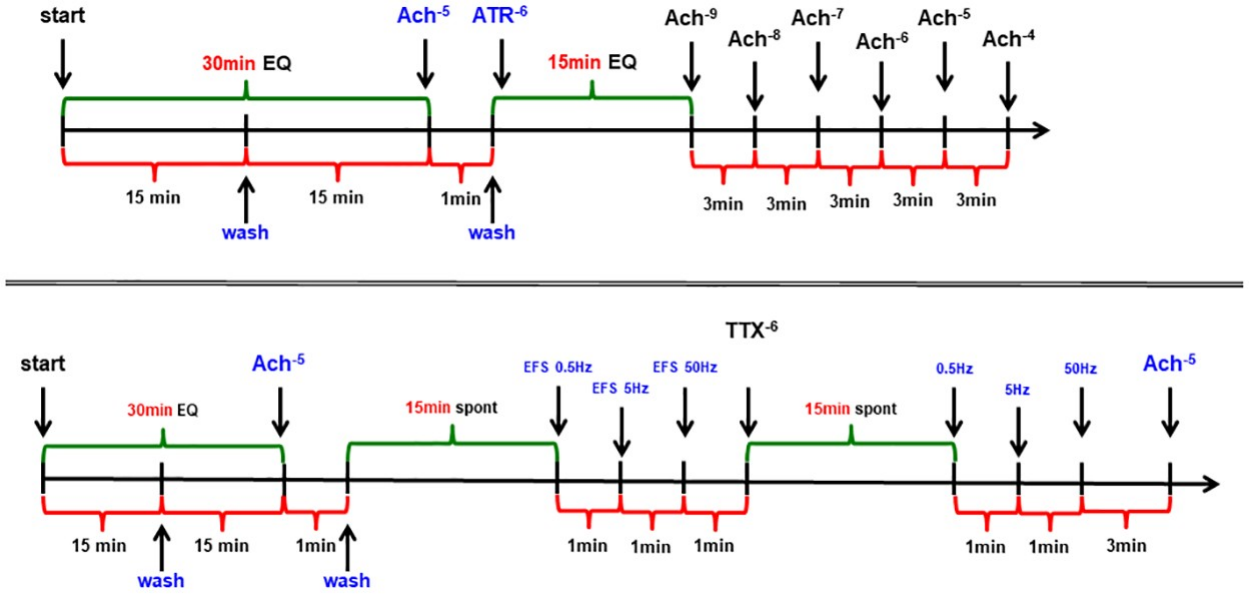
Scheme of the intestinal contractility *in vitro* study.

To investigate intestinal contractility, duodenal and middle jejunum segments 15 mm long were immediately collected from piglets upon euthanasia and placed in cold Krebs-Henseleit buffer (in mM: NaCl 18, KCl 4.7, KH_2_PO_4_ 1.2, MgSO_4_ 1.2, CaCl_2_ 1.25, NaHCO_3_ 25, glucose 11), as described before [12]. The intestinal sections were then put in vertical position in 25 ml organ bath chambers (Letica Scientific Instruments, Spain) filled with Krebs-Henseleit solution (37°C, pH 7.4) and continuously saturated with carbogen (95% O_2_, 5% CO_2_). The intestinal segments were equilibrating for 30 minutes to recover spontaneous activity, during which the solution in the chambers was changed once after 15 minutes (Fig 1). The tissues were attached to isotonic transducers (Letica Scientific Instruments, Spain) under a load of 1.0 g that were coupled with a PowerLab recording system (ADInstruments, Sydney, Australia). Next, the segments were treated with 10^−5^ M ACh for 1 minute. The tissues were then washed and allowed to equilibrate. Finally, the spontaneous or ACh-stimulated contractility as the response to growing and cumulative doses of ACh (from 10^−9^ to 10^−4^ M) was noted. Half of randomly selected jejunal segments were pre-treated with atropine.

To examine the effect of obestatin on neural contractions, the electric field stimulation (EFS) was used [12]. After equilibration, EFS (EXP-ST-01, Experimetria, Budapest, Hungary, voltage 90 V, duration 10 seconds) at three frequencies: 0.5, 5 and 50 Hz, with 1-minute intervals between each pulse was performed (Fig 1). The remaining half of jejunal sections were pre-treated with TTX. Each experiment was terminated with the administrations of 10^−5^ M ACh to check the viability of the tissue, followed by 10^−5^ M isoproterenol to control its relaxation.

### Histology of the intestinal muscularis layer

For histometrical analysis, the samples of duodenal and middle jejunum segments (15 mm long) were collected and immediately fixed in a 10% neutral formalin solution for 24 hours [19]. Briefly, the samples were routinely embedded in paraffin and cut into 4.5 μm sections and applied to silane-treated slides. Next, the sections were immersed in xylene for dewaxing and then in descending grades of alcohol for rehydration. The segments were routinely stained using hematoxylin and eosin. For each intestinal section three slides were randomly selected and 30 measurements of the muscularis layer were performed using light microscope (Axioskop 40, Zeiss, Germany) coupled with a computer with image analysis software (Axio Vision 4.2 Release, Zeiss, Germany).

### Immunodetection of the muscarinic ACh receptors 1 and 2 (M1 and M2 receptors)

Immunoblotting of muscarinic ACh receptors 1 and 2 (M1 and M2 receptors) was performed in segments of the middle part of jejunum frozen immediately after collection at -80°C, according to the procedure described earlier [19]. Before being analyzed, the samples were thawed and about 0.5 g of tissues from each experimental group were homogenized in RIPA buffer (50 mM Tris, pH 7.5, 150 mM NaCl, 1 mM EDTA, 1% NP-40, 0.25% Na-deoxycholate and 1 mM PMSF) with the addition of protease inhibitor and phosphatase inhibitor cocktails (Sigma-Aldrich, Germany). To ensure complete cell lysis, the samples were incubated on ice for 30 minutes and then centrifuged for 30 minutes at 14,000 rpm, and supernatants with extracted proteins were collected. The concentration of protein was determined using Bio-Rad Protein Assay Dye Reagent following the producer’s instructions (Bio-Rad Laboratories Inc., Hercules, CA, USA). Samples containing 50 μg of proteins were resolved using SDS-PAGE and transferred to a PVDF membrane (Sigma-Aldrich, Germany). For immunostaining, the blotted membranes were blocked with 5% nonfat dry milk in TBST (20 mM Tris-HCl, 500 mM NaCl, 0.5% Tween 20). The membranes were then probed with antibodies against M1 and M2 receptors (Abcam 180636, Abcam, cat. no: ab188891; 1:250 dilution, respectively), or b-actin (Santa Cruz Biotechnologies, Inc., cat. no: sc-47778, 1:1000 dilution) at 4°C overnight. After being washed (15 minutes, three times), the membranes were incubated with appropriate IR fluorophores-conjugated secondary antibodies: IR Dye 680CW or IR Dye 800CW (at 1:5000 dilution). Following the incubation, the membranes were washed three times in TBST. For the analysis of protein expression an Odyssey Infrared Imaging System was used (LI-COR Biosciences, USA) with scan resolution set at 169 lm, and the intensity at 4. Quantification of the integrated optical density (IOD) was performed.

### Statistical analysis

Results are expressed as means ± SEM with significance defined as *p* ˂ 0.05. A one-way ANOVA followed by the Tukey post-hoc test or Kruskal-Wallis test, Mann-Whitney or an unpaired t test was used to assess the statistical differences between the groups. Also, two-way ANOVA with Bonferroni’s multiple comparisons post-hoc test was done. In all investigated parameters, we observed the effect of the place (duodenum vs. jejunum) or the effect of the obestatin but we did not observe any interactions between the place of action and obestatin in different doses. All analyses were performed using GraphPad Prism version 4.0b (GraphPad Software Inc, San Diego, CA, USA).

## Results

### Obestatin concentration in blood plasma

Obestatin concentration (pg/mL) in control piglets was stable in the measured period between 8^th^ and 22^nd^ day of life. Administration of obestatin at a dose of 2 μg/kg BW (group O2) did not affect peptide concentration neither 30 nor 60 minutes after its administration as compared to C group. Administration of obestatin at the dose of 10 μg/kg BW (group O10) resulted in an elevated peptide concentration on days 12, 14, 18, 20 and 22 at 30 minutes after the administration, and in all measured time points from 8^th^ till 22^nd^ day of life at 60 minutes after the administration. Administration of the highest dose of obestatin (15 μg/kg BW (group O15)) significantly elevated obestatin concentration on days 12, 14 and 22 at 30 minutes after peptide administration. After 60 minutes from obestatin administration, the concentration of peptide in group O15 increased significantly in all measured time points in comparison to control animals (Table 1).

**Table 1:** The effect of enteral obestatin administration on obestatin concentration [pg/mL] in the blood plasma of piglets.

Group	Day 8	Day 10	Day 12	Day 14	Day 16	Day 18	Day 20	Day 22	*P*
	30 min after obestatin administration								
C	334 ± 32	357 ± 32	329 ± 28^a^	325 ± 31^a^	344 ± 39	325 ± 29^a^	341 ± 38^a^	384 ± 45^a^	*0*.*9388*
O2	362 ± 23^A^	369 ± 26^A^	372 ± 20^a^^b^^A^	381 ± 15^a^^b^^A^	388 ± 36^A^^B^	376 ± 13^a^^b^^A^	384 ± 13^a^^b^^A^^B^	481 ± 17^a^^b^^B^	*0*.*0084*
O10	413 ± 11^A^	431 ± 13^A^	422 ± 23^b^^A^	444 ± 18^b^^A^^B^	432 ± 13^A^	441 ± 26^b^	449 ± 15^b^^A^^B^	522 ± 25^b^^B^	*0*.*0066*
O15	405 ± 26	394 ± 22	422 ± 30^b^	435 ± 41^b^	420 ± 64	403 ± 31^a^^b^	439 ± 72^a^^b^	505 ± 36^b^	*0*.*0902*
*P*	*0*.*1056*	*0*.*1951*	*0*.*0393*	*0*.*0020*	*0*.*1840*	*0*.*0216*	*0*.*0101*	*0*.*0231*	
	60 min after obestatin administration								
C	317 ± 20^a^	313 ± 32^a^	364 ± 35^a^	348 ± 33^a^	347 ± 40^a^	322 ± 28^a^	351 ± 30^a^	381 ± 28^a^	*0*.*7560*
O2	370 ± 23^a^^A^	380 ± 25^a^^b^^A^	371 ± 22^a^^A^	389 ± 17^a^^A^	389 ± 32^a^^A^	378 ± 14^a^^b^^A^	388 ± 10^a^^A^	501 ± 9^a^^B^	*0*.*0060*
O10	475 ± 22^b^*^A^	498 ± 31^b^^A^**	491 ± 19^b^^A^*	502 ± 47^b^^A^*	493 ± 23^b^^A^*	498 ± 29^b^^A^	504 ± 11^b^^A^**	602 ± 23^b^^B^*	*0*.*0002*
O15	450 ± 53^b^^A^	490 ± 49^b^^A^**	494 ± 74^b^^A^^B^	515 ± 42^b^^A^^B^*	503 ± 49^b^^A^^B^*	485 ± 26^b^^A^**	487 ± 45^b^^A^	594 ± 12^b^^B^**	*0*.*0018*
*p*	*<0*.*0001*	*0*.*0005*	*0*.*0013*	*<0*.*0001*	*0*.*0014*	*0*.*0003*	*0*.*0003*	*<0*.*0001*	

Piglets fed milk formula with intra-stomach administrations of obestatin: 2 μg/kg body weight (BW)–O2, 10 μg/kg BW–O10, 15 μg/kg BW–O15, or 0.9% NaCl–C, every 8 hours. Results are presented as means ± SEM.

^a,b^- indicates statistical differences between groups within the day.

^A,B^- indicates statistical differences between following days within one group.

*- indicates statistical differences between the time points within one group.

### Effect of obestatin on spontaneous contractility

**Fig 2:**
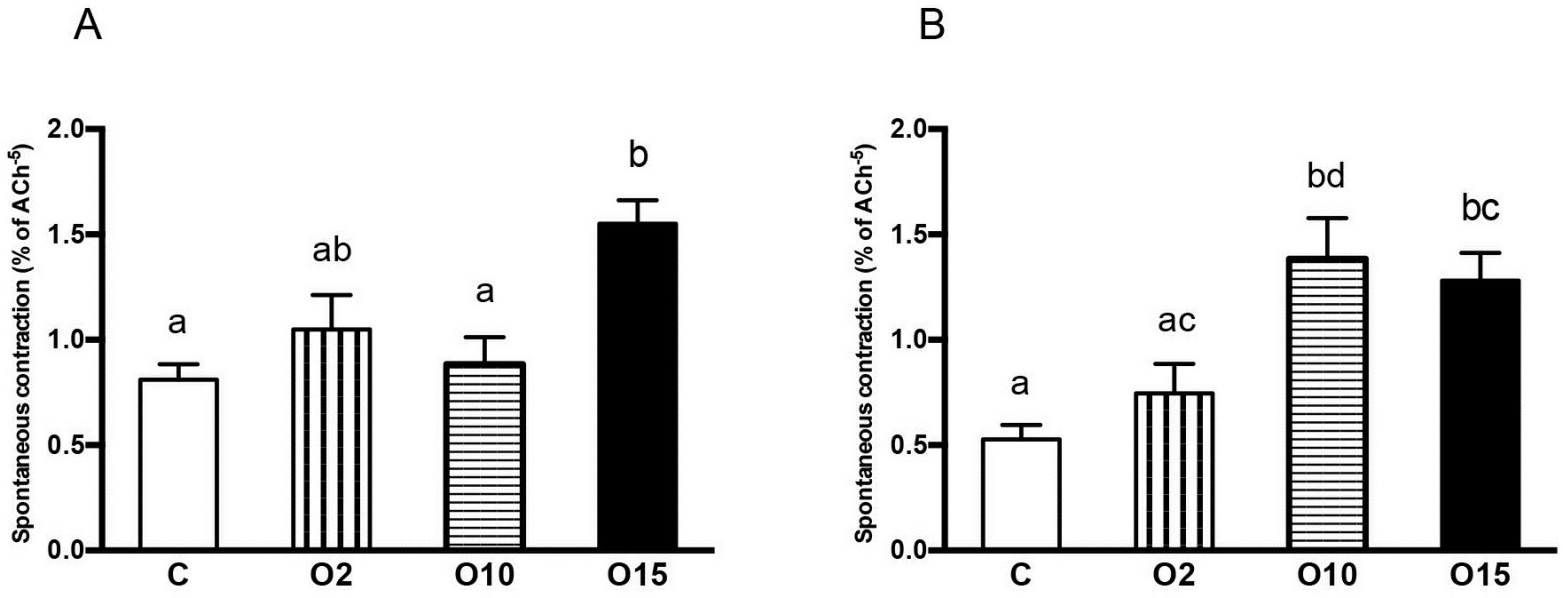
Effect of obestatin on the amplitude [mm] of spontaneous intestinal contractility of the duodenum (A) and middle jejunum (B) in newborn piglets. The amplitude of spontaneous contractions (determined as the percentage of response to acetylcholine chloride (10^−5^ M ACh)) in duodenal and middle jejunum segments from piglets fed milk formula with intra-stomach administrations of obestatin (2 μg/kg body weight (BW)–O2, 10 μg/kg BW–O10, 15 μg/kg BW–O15) or 0.9% NaCl–C, every 8 hours. Results are presented as means ± SEM. ^a,b,c^- indicates statistical differences between groups.

The amplitude and frequency of contraction were analyzed to investigate the effects of obestatin on spontaneous intestinal contractility. In both intestinal segments studied, significant increases in the frequency of contractions were observed in C and O15 animals (Table 2). The amplitude of contractions in the duodenal segment was significantly higher in O15 piglets than in C ones. Moreover, the effect of obestatin dose was observed in the amplitude of contractions in piglets from groups O10 and O15. In the middle jejunum, a significant increase in the amplitude of contractions was observed in C piglets in comparison to O10 and O15 groups (Fig 2).

**Table 2:** The effect of enteral obestatin administration on the frequency of spontaneous contractions [determined as the percentage of response to acetylcholine chloride (10^−5^ M ACh)] in duodenal and middle jejunum strips from piglets.

	C	O2	O10	O15	*P*
Duodenum					
Mean Frequency	16.4 ± 1.9^a^	20.4 ± 0.9^a^^b^	20.4 ± 1.05^a^^b^	22.6 ± 1.3^b^	*0*.*0469*
Middle jejunum					
Mean Frequency	17.8 ± 3.4^a^	18.2 ± 1.6^a^	17.3 ± 2.0^a^	27.9 ± 1.1^b^	*0*.*0160*

Piglets fed milk formula with intra-stomach administrations of obestatin: 2 μg/kg body weight (BW)–O2, 10 μg/kg BW–O10, 15 μg/kg BW–O15, or 0.9% NaCl–C, every 8 hours. Results are presented as means ± SEM.

^a,b,c^- indicates statistical differences between groups.

#### Effect of obestatin on EFS

**Fig 3:**
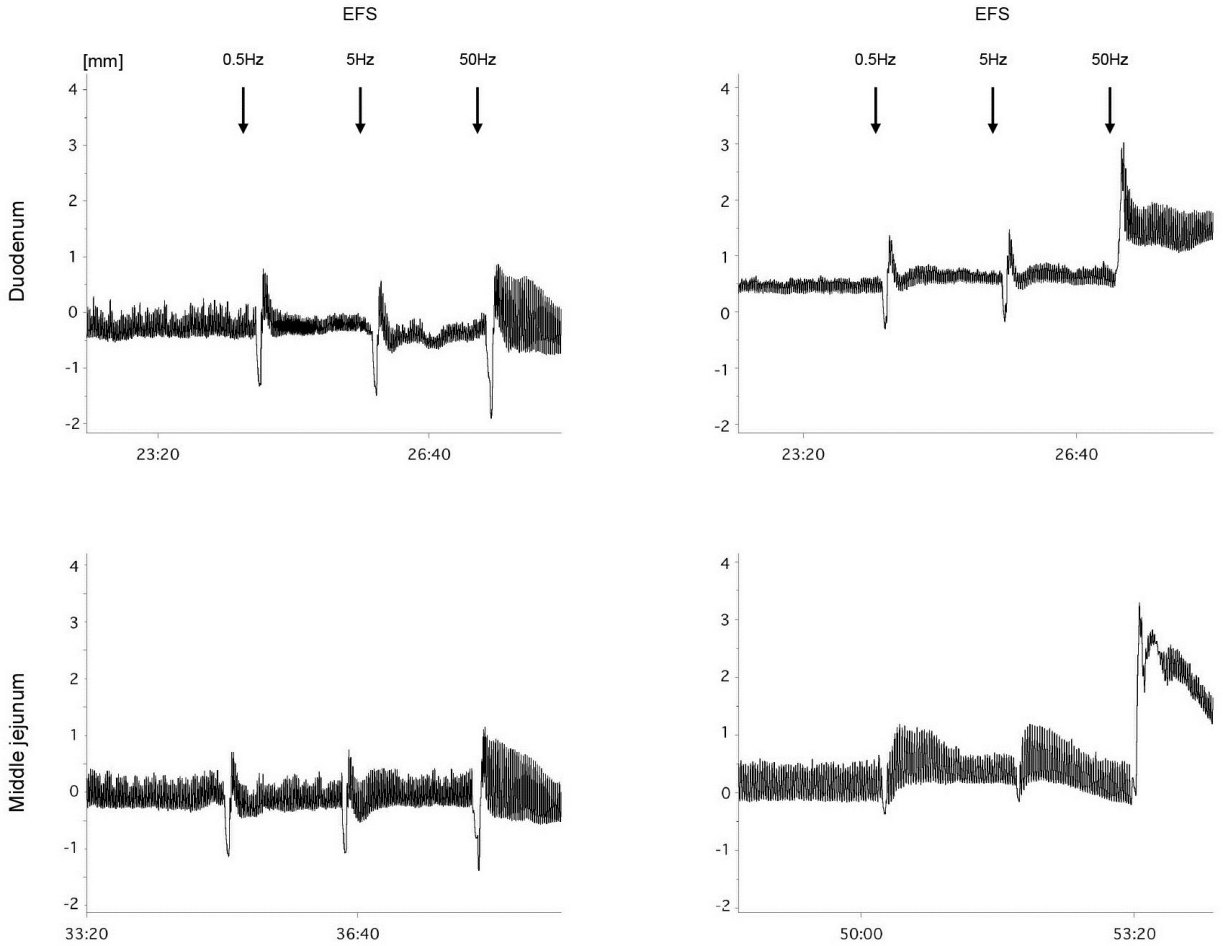
Representative trace of response to electrical field stimulation (EFS) [mm] in duodenum and middle jejunum of newborn piglets. EFS (0.5 Hz, 5 Hz, 50 Hz) in duodenal (upper panel) and middle jejunum (lower panel) segments from piglets fed milk formula with intra-stomach administrations of 0.9% NaCl–C, every 8 hours (left graphs) and obestatin (15 μg/kg BW–O15) (right graphs).

EFS impulses (0.5, 5, 50 Hz) resulted in a frequency-dependent increase in the amplitude of contractions in both the duodenal and middle jejunum segments. In the duodenal segments, significant differences in the amplitude of contractions were observed between piglets treated with different doses of obestatin, following EFS at 0.5 and 5 Hz. A significant increase in the amplitude of contractions was observed in O15 piglets in comparison to C group, following EFS at 50 Hz. In the middle jejunum segments, treatment with obestatin at a dose of 15 μg/kg BW (group O15) resulted in a significant increase in the amplitude of contractions as compared to C group, following EFS using 0.5 and 50 Hz frequencies. Also, the middle jejunum segments treated with obestatin at a dose of 15 μg/kg BW (group O15) displayed significantly increased amplitude of contractions in comparison to that observed in the low dose obestatin group (O2), following EFS of 5 Hz. Following pre-incubation with TTX, the effects of obestatin were blocked and contractile responses were similar to those observed in saline-treated preparations (Table 3, Fig 3).

**Table 3:** The effect of enteral obestatin administration on the electrical field stimulation (EFS)—Evoked amplitude of contraction [mm] in the presence or absence of Tetrodotoxin (TTX (10^−6^ M)) in duodenal and middle jejunum strips from piglets.

		Basal			TTX^-6^		
		0.5 Hz	5 Hz	50 Hz	0.5 Hz	5 Hz	50 Hz
Duodenum	C	1.54 ± 0.23^a^^b^	1.63 ± 0.27^a^^b^	1.88 ± 0.21^a^	0.77 ± 0.14**	0.80 ± 0.15*	1.50 ± 0.20
	O2	2.53 ± 0.31^a^	2.78 ± 0.35^a^	2.95 ± 0.28^a^^c^	0.48 ± 0.11**	0.54 ± 0.11**	0.82 ± 0.21***
	O10	1.08 ± 0.26^b^	1.25 ± 0.32^b^	1.20 ± 0.29^a^^d^	0.56 ± 0.16	0.59 ± 0.15	0.86 ± 0.22
	O15	2.34 ± 0.30^a^	2.74 ± 0.37^a^	3.38 ± 0.36^b^^c^	0.40 ± 0.09***	0.46 ± 0.01***	0.77 ± 0.22****
	*P*	*0*.*0045*	*0*.*0068*	*0*.*0021*	*0*.*3667*	*0*.*4610*	*0*.*1196*
Middle jejunum	C	1.04 ± 0.22^a^	1.31 ± 0.28^a^^b^	1.83 ± 0.32^a^	0.43 ± 0.16*	0.49 ± 0.16*	0.78 ± 0.21*
	O2	0.53 ± 0.26^a^^c^	0.70 ± 0.35^a^	1.22 ± 0.59^a^	0.17 ± 0.07	0.20 ± 0.08	0.31 ± 0.17
	O10	1.75 ± 0.18^a^^d^	1.75 ± 0.18^a^^b^	1.49 ± 0.30^a^	0.28 ± 0.10****	0.30 ± 0.12****	0.45 ± 0.15**
	O15	2.47 ± 0.23^b^^d^	2.74 ± 0.37^b^	3.76 ± 0.29^b^	0.46 ± 0.23****	0.34 ± 0.11****	0.61 ± 0.27****
	*P*	*<0*.*0001*	*0*.*0023*	*0*.*0003*	*0*.*4393*	*0*.*5545*	*0*.*3511*

Piglets fed milk formula with intra-stomach administrations of obestatin: 2 μg/kg body weight (BW)–O2, 10 μg/kg BW–O10, 15 μg/kg BW–O15, or 0.9% NaCl–C, every 8 hours. Results are presented as means ± SEM.

^a,b^- indicates statistical differences between groups in the absence of tetrodotoxin (TTX).

*-indicates statistical differences between control and obestatin group in the presence of TTX.

#### Effect of obestatin on ACh-stimulated contractility

Rising cumulative doses of ACh (from 10^−9^ to 10^−4^ M) resulted in a dose-dependent increase in the amplitude of intestinal contractility *in vitro* (Table 4). It was observed that the responsiveness to rising doses of ACh is dependent on the treatment and the studied segment of the intestine. In the duodenum, no significant differences in the responsiveness to ACh were observed between the different treatment groups until the addition of 10^−4^ M ACh. After the addition of 10^−4^ M ACh, a significant increase in the responsiveness of the duodenal segments was observed in C group in comparison to O15 piglets. In contrast, a significant increase in the responsiveness was observed in the middle jejunum segments of all treatment groups, starting from the addition of 10^−9^ M ACh. As compared to control piglets, a significant increase in the amplitude of contractions was observed in the O15 group following the addition of 10^−9^ M ACh, and in the O10 and O15 groups following the addition of 10^−6^ and 10^−4^ M ACh, respectively. Pretreatment with atropine resulted in a significant decrease in the responsiveness in all treatment groups, and in both intestinal segments studied. In the middle jejunum, treatment with obestatin at doses of 10 μg/kg BW (group O10) and 15 μg/kg BW (group O15) resulted in a significantly higher responsiveness after blockage with atropine in comparison to that observed in C piglets.

**Table 4:** The effect of enteral obestatin administration on the amplitude [mm] of acetylcholine chloride (ACh) stimulated or atropine treated contractions in duodenal and middle jejunum strips from piglets.

Duodenum							
	ACh^-9^	ACh^-8^	ACh^-7^	ACh^-6^	ACh^-5^	ACh^-4^	ATR^-6^
C	4.9 ± 0.6	5.0 ± 0.6	5.3 ± 0.6	5.3 ± 0.5	9.1 ± 0.3	10.5 ± 0.4^a^	0.77 ± 0.1
O2	6.2 ± 0.3	7.0 ± 0.7	7.1 ± 0.7	7.4 ± 0.9	11.2 ± 0.7	14.1 ± 1.2^a^^b^	1.26 ± 0.2
O10	5.9 ± 0.9	6.0 ± 0.8	6.1 ± 0.9	6.3 ± 0.9	8.2 ± 0.9	12.3 ± 1.1^a^^b^	0.97 ± 0.1
O15	5.3 ± 0.5	5.4 ± 0.4	5.9 ± 0.6	6.7 ± 0.8	9.6 ± 0.6	15.1 ± 0.4^b^	1.0 ± 0.1
*P*	*0*.*1371*	*0*.*1252*	*0*.*1625*	*0*.*5168*	*0*.*2682*	*0*.*0233*	*0*.*2057*
Middle jejunum							
	ACh^-9^	ACh^-8^	ACh^-7^	ACh^-6^	ACh^-5^	ACh^-4^	ATR^-6^
C	3.8 ± 0.5^a^	4.3 ± 0.5	4.7 ± 0.7	3.8 ± 0.4^a^	7.8 ± 0.9	8.6 ± 0.9^a^	0.71 ± 0.1^a^
O2	5.9 ± 0.8^a^^b^	6.0 ± 0.7	6.1 ± 0.8	6.3 ± 0.9^a^^b^	8.0 ± 0.9	12.3 ± 1.0^a^^b^	0.97 ± 0.1^a^^b^
O10	3.9 ± 0.8^a^^b^	4.6 ± 0.9	4.8 ± 0.9	7.0 ± 0.9^b^	10.1 ± 0.7	12.5 ± 0.9^b^	1.10 ± 0.2^b^
O15	6.6 ± 0.7^b^	6.7 ± 0.7	6.8 ± 0.6	7.3 ± 0.8^b^	9.0 ± 0.8	13.7 ± 0.8^b^	1.20 ± 0.1^b^
*P*	*0*.*0253*	*0*.*1108*	*0*.*1882*	*0*.*0214*	*0*.*2463*	*0*.*0038*	*0*.*0001*

Piglets fed milk formula with intra-stomach administrations of obestatin: 2 μg/kg body weight (BW)–O2; 10 μg/kg BW–O10; 15 μg/kg BW–O15, or 0.9% NaCl–C, every 8 hours. Results are presented as means ± SEM.

^a,b^- indicates statistical differences between groups.

### Obestatin and thickness of muscularis layer

In the duodenum, the enteral treatment with obestatin in all studied doses (O2, O10, O15) decreased the thickness of muscularis layer in comparison to control animals (C). In the middle jejunum the decrease in the thickness of muscularis layer was observed for groups O10 and O15 (Table 5).

**Table 5:** Effect of intra-stomach obestatin administration on the thickness of muscularis layer [μm] in duodenum and middle jejunum of piglets.

	C	O2	O10	O15	*P*
Duodenum					
Thickness of muscularis layer	195 ± 9^a^	106 ± 11^b^	107 ± 8^b^	89 ± 8^b^	*<0*.*0001*
Middle jejunum					
Thickness of muscularis layer	107 ± 5^a^	88 ± 9^a^	63 ± 7^b^	66 ± 4^b^	*<0*.*0001*

Piglets fed milk formula with intra-stomach administrations of obestatin: 2 μg/kg body weight (BW)–O2, 10 μg/kg BW–O10, 15 μg/kg BW–O15, or 0.9% NaCl–C, every 8 hours. Results are presented as means ± SEM.

^a,b^- indicates statistical differences between the groups

### Effect of obestatin on muscarinic ACh receptors (M1 and M2 receptors)

**Fig 4:**
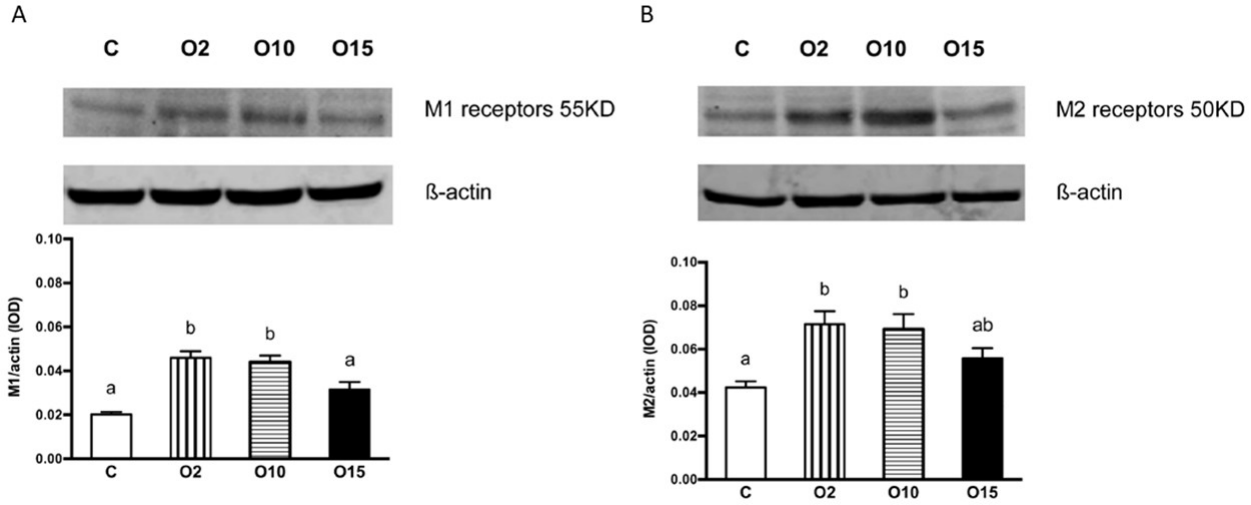
Western blot analysis of M1 receptor expression [IOD] in the mucosa of middle jejunum segments in newborn pigs and optical density of the M1 receptor b-actin ratio in the study groups (A). Western blot analysis of M2 receptor expression [IOD] in the mucosa of middle jejunum segments in newborn pigs and optical density of the M2 receptor b-actin ratio in the study groups (B). Piglets fed milk formula with intra-stomach administrations of obestatin (2 μg/kg body weight (BW)–O2, 10 μg/kg BW–O10, 15 μg/kg BW–O15) or 0.9% NaCl–C, every 8 hours. Results are presented as means ± SEM. ^a,b,c^- indicates statistical differences between groups.

The enteral administration of obestatin at doses of 2 μg/kg BW (group O2) and 10 μg/kg BW (group O10), significantly increased the cytoplasmic expression of both M1 and M2 receptors in the middle jejunum, whereas treatment with the highest dose (15 μg/kg BW; group O15) had no significant effect (Fig 4).

## Discussion

The present study was focused on the effect of obestatin administered enterally to piglets fed milk formula on the contractility of whole-thickness intestinal preparations. Milk formulas are introduced to the swine production industry in order to feed underprivileged piglets or large numbers of piglets. Previous studies have shown that due to the lack of growth factors and hormones in formulas, formula-fed piglets display a marked delay in the intestinal maturation during the pivotal suckling period [14]. The experimental model used in the present study was based on the assumption that gastric and duodenal proteolysis in the newborns is minimal, and thus allow for sufficient absorption of intact milk peptides that are crucial for the GIT development during the first days of life [14–16]. In our study, rat-origin obestatin was enterally administered to piglets and its increased concentration in the blood circulation was observed. It is worth mentioning that obestatin is well conserved among species and there is 87% of homology in the amino acid structure between rat and pig peptide [17].

Previous obestatin studies revealed controversial data showing an inhibitory effect [10] on intestinal contractility *in vitro*. It should be mentioned that these results were obtained from experiments conducted on adult animals and isolated muscular strips. In our recent study [12] it was demonstrated that intestinal contractility of rats, studied *in vitro*, is affected by obestatin and the effects are dependent on the age of the rats, the segment of the intestine and the methods used for preparation of the intestinal segments. We suspect that the removal of mucosa and submucosa layers may have a significant effect on neuromuscular transmission. Thus, in our previous as well as current experiments, we have studied whole-thickness intestinal preparations, instead of just a single muscular layer. Moreover, Mondal et al. [18] proved the important role of the mucosa for ghrelin-induced gastric contractions, suggesting the existence of specialized interneurons that may be responsible for a signal transmission from ghrelin receptors located in the mucosa to the myenteric plexus. This may be true also for obestatin.

Interestingly, in our previous study on obestatin administered enterally to suckling rats, starting from 14^th^ till the 21^st^ day of life, we have observed a significant decrease in the amplitude of spontaneous and EFS-evoked contractions in animals treated with the peptide [19]. On the other hand, in the present study we reported a significant increase in the intestinal contractility after the treatment with the highest dose of rat-origin obestatin. These results strongly suggest that intestinal neurotransmission is species-specific. Moreover, our results confirmed previous observations in suckling rats [12,19] that the neonatal intestine is sensitive to obestatin. This seems reasonable from a physiological point of view considering that significant amounts of obestatin are found in swine (Woliński et al., unpublished data), rat and human milk [20,21], and therefore enter the gut lumen during each feeding. For the same reason, it seems logical that the fully developed and functional intestine of adults may react differently to obestatin. It should be also underlined that in our study on neonatal rats [12], animals were reared by their mothers and additionally supplemented with obestatin, whereas in the pig study, animals were fed milk formula without this peptide, thus intra-stomach administration was the only source of obestatin.

The mechanism by which obestatin influences intestinal contractility is unknown. Because cognate receptor(s) for obestatin remains elusive, the interpretation of available data is difficult and conducting this type of research even more complicated. The results of the present as well as previous studies [12,19], and the fact that obestatin-induced intestinal contractility was susceptible to TTX and atropine, could suggest that intrinsic neural activity or the activation of muscarinic receptors (by releasing the intrinsic neurotransmitter, ACh) mediate the intestinal response to obestatin. However, immunoblotting of M1 and M2 receptors in the middle jejunum showed no differences between C and O15 groups suggesting that increased contractility observed in O15 group is not dependent on muscarinic receptors. Further studies on the number of cholinergic nerves in animals treated with obestatin could fully confirmed this observation.

In the present study we demonstrated the obestatin effect on intestine of 7-day-old piglets fed milk formula. A limited number of studies on intestinal contractility and the use of EFS in pigs fed formula is available. In the study by Woliński et al. [22], the decrease in the responsiveness to ACh in 7-day-old piglets fed milk formula in comparison to sow-reared ones was shown. Moreover, Zhang [23] reported a decreased number of noradrenergic nerve endings in the enteric nervous system of formula-fed piglets. Electrogastrography (EGG) performed on human newborns demonstrated a higher percentage of normal slow waves in breast milk-fed babies than in those formula-fed [24]. These findings may suggest that the increase in intestinal contractility following treatment with obestatin observed in the current study is a desired effect, which makes the milk formula less harmful to the neonate. Unlike to our previous study on suckling rats [19], the enteral administration of obestatin in the higher doses (O10, O15) to suckling piglets increased intestinal contractility and decreased the thickness of muscularis layer. Based on the presented results it can be speculated that stronger intestinal contractility could influence transit of nutrients resulting in a shorter availability of nutrients from the intestinal lumen, thereby preventing muscle overgrowth. Further studies are required in order to elucidate the effect of obestatin on the developmental process of the GIT in suckling mammals.

## Conclusions

In conclusion, our study characterized intestinal contractility in neonatal piglets fed milk formula and documented the importance of enteral obestatin administration in the regulation of this process.

## Supporting information

S1 Fig Western blot analysis of M1 receptors expression in the mucosa of middle jejunum segments in newborn pigs **(A, B)**. Western blot analysis of M2 receptors expression in the mucosa of middle jejunum segments in newborn pigs **(C, D)**. Original images of PVDF membranes incubated with a set of antibodies: primary antibody rabbit anti-M1R or anti-M2R for M1 and M2 receptors, respectively, and secondary antibody conjugated with IRDye^®^ 800CW (green fluorescence), or primary antibody mouse anti-beta-actin and secondary antibody conjugated with IRDye^®^ 680CW (red fluorescence) (A, C). Original images showing bands representing only green channel (after anti-M1R or anti-M2R antibodies, respectively) converted into a black and white image (B, D). The scans performed using Odyssey Infrared Imaging System (LI-COR Biosciences). Piglets fed milk formula with intra-stomach administrations of obestatin (2 μg/kg body weight (BW)–O2, 10 μg/kg BW–O10, 15 μg/kg BW–O15) or 0.9% NaCl–C, every 8 hours, M–marker.
(PDF)
