# Correction: The effects of intra-stomach obestatin administration on intestinal contractility in neonatal piglets fed milk formula

**DOI:** 10.1371/journal.pone.0231945

**Published:** 2020-04-14

**Authors:** Monika Słupecka-Ziemilska, Paulina Szczurek, Maria Boryczka, Małgorzata Gajewska, Piotr Wychowański, Atsukazu Kuwahara, Ikuo Kato, Żaneta Dzięgelewska, Jarosław Woliński

The first affiliation is missing for the first author. Monika Słupecka-Ziemilska’s first affiliation is: Department of Human Epigenetics, Mossakowski Medical Research Centre, Polish Academy of Sciences, Warsaw, Poland.

The correct affiliations are as follows:

Monika Słupecka-Ziemilska^1,2^, Paulina Szczurek^3^, Maria Boryczka^2^, Małgorzata Gajewska^4^, Piotr Wychowański^5^, Atsukazu Kuwahara^6^, Ikuo Kato^7^, Żaneta Dzięgelewska^4^, Jarosław Woliński^2^

**1** Department of Human Epigenetics, Mossakowski Medical Research Centre, Polish Academy of Sciences, Warsaw, Poland, **2** Department of Animal Physiology, Polish Academy of Sciences, The Kielanowski Institute of Animal Physiology and Nutrition, Jabłonna, Poland, **3** Department of Animal Nutrition and Feed Sciences, National Research Institute of Animal Production, Balice, Poland, **4** Department of Physiological Sciences, Faculty of Veterinary Medicine, Warsaw University of Life Sciences, Warsaw, Poland, **5** Department of Dental Surgery, Medical University of Warsaw, Warsaw, Poland, **6** Laboratory of Physiology, Institute for Environmental Sciences & Graduate School of Nutritional and Environmental Sciences, University of Shizuoka, Shizuoka, Japan, **7** Department of Medical Biochemistry, Kobe Pharmaceutical University, Kobe, Japan.

## References

[1] Słupecka-Ziemilska M, Szczurek P, Boryczka M, Gajewska M, Wychowański P, Kuwahara A, et al (2020) The effects of intra-stomach obestatin administration on intestinal contractility in neonatal piglets fed milk formula. PLoS ONE 15(3): e0230190 10.1371/journal.pone.0230190 32203550 PMC7089538

