# Supplementary figures and images for: The effects of intra-stomach obestatin administration on intestinal contractility in neonatal piglets fed milk formula
Source: PLoS One. 2020 Mar 23;15(3):e0230190. doi: 10.1371/journal.pone.0230190 (PMC7089538; doi:10.1371/journal.pone.0230190)

**A**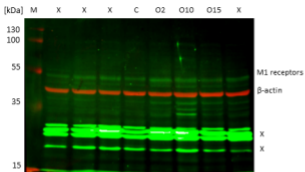**B**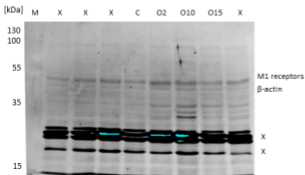**C**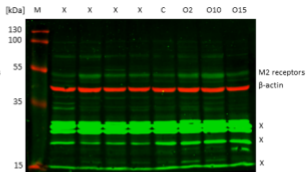**D**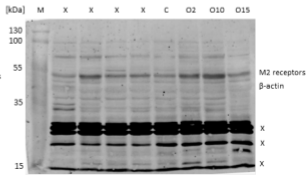

Supplement: S1 Fig — Western blot analysis of M1 receptors expression in the mucosa of middle jejunum segments in newborn pigs (A, B). Western blot analysis of M2 receptors expression in the mucosa of middle jejunum segments in newborn pigs (C, D). Original images of PVDF membranes incubated with a set of antibodies: primary antibody rabbit anti-M1R or anti-M2R for M1 and M2 receptors, respectively, and secondary antibody conjugated with IRDye® 800CW (green fluorescence), or primary antibody mouse anti-beta-actin and secondary antibody conjugated with IRDye® 680CW (red fluorescence) (A, C). Original images showing bands representing only green channel (after anti-M1R or anti-M2R antibodies, respectively) converted into a black and white image (B, D). The scans performed using Odyssey Infrared Imaging System (LI-COR Biosciences). Piglets fed milk formula with intra-stomach administrations of obestatin (2 μg/kg body weight (BW)–O2, 10 μg/kg BW–O10, 15 μg/kg BW–O15) or 0.9% NaCl–C, every 8 hours, M–marker. (PDF) [file pone.0230190.s001.pdf]
